# Maximizing Upconversion Luminescence of Co-Doped CaF₂:Yb, Er Nanoparticles at Low Laser Power for Efficient Cellular Imaging

**DOI:** 10.3390/molecules29174177

**Published:** 2024-09-03

**Authors:** Neha Dubey, Sonali Gupta, Sandeep B. Shelar, K. C. Barick, Sudeshna Chandra

**Affiliations:** 1 Department of Chemistry, Sunandan Divatia School of Science, SVKM’s NMIMS (Deemed To-Be) University, Mumbai 400056, India; 2 Chemistry Division, Bhabha Atomic Research Centre, Trombay, Mumbai 400085, India; 3 Homi Bhabha National Institute, Anushaktinagar, Mumbai 400094, India; 4 Hanse-Wissenschaftskolleg–Institute for Advanced Study (HWK), Lehmkuhlenbusch 4, 27753 Delmenhorst, Germany

**Keywords:** upconversion, nanoparticles, CaF_2_, lanthanide doping, luminescence, cellular imaging

## Abstract

Upconversion nanoparticles (UCNPs) are well-reported for bioimaging. However, their applications are limited by low luminescence intensity. To enhance the intensity, often the UCNPs are coated with macromolecules or excited with high laser power, which is detrimental to their long-term biological applications. Herein, we report a novel approach to prepare co-doped CaF_2_:Yb^3+^ (20%), Er^3+^ with varying concentrations of Er (2%, 2.5%, 3%, and 5%) at ambient temperature with minimal surfactant and high-pressure homogenization. Strong luminescence and effective red emission of the UCNPs were seen even at low power and without functionalization. X-ray diffraction (XRD) of UCNPs revealed the formation of highly crystalline, single-phase cubic fluorite-type nanostructures, and transmission electron microscopy (TEM) showed co-doped UCNPs are of ~12 nm. The successful doping of Yb and Er was evident from TEM–energy dispersive X-ray analysis (TEM-EDAX) and X-ray photoelectron spectroscopy (XPS) studies. Photoluminescence studies of UCNPs revealed the effect of phonon coupling between host lattice (CaF_2_), sensitizer (Yb^3+^), and activator (Er^3+^). They exhibited tunable upconversion luminescence (UCL) under irradiation of near-infrared (NIR) light (980 nm) at low laser powers (0.28–0.7 W). The UCL properties increased until 3% doping of Er^3+^ ions, after which quenching of UCL was observed with higher Er^3+^ ion concentration, probably due to non-radiative energy transfer and cross-relaxation between Yb^3+^-Er^3+^ and Er^3+^-Er^3+^ ions. The decay studies aligned with the above observation and showed the dependence of UCL on Er^3+^ concentration. Further, the UCNPs exhibited strong red emission under irradiation of 980 nm light and retained their red luminescence upon internalization into cancer cell lines, as evident from confocal microscopic imaging. The present study demonstrated an effective approach to designing UCNPs with tunable luminescence properties and their capability for cellular imaging under low laser power.

## 1. Introduction

Upconversion nanoparticles (UCNPs) are considered a powerful tool in cellular imaging owing to their ability to convert low-energy near-infrared (NIR) light into higher-energy visible light, which enables deeper penetration, reduced background autofluorescence, and distinguishing photosensitive traits. In UCNPs, Ln^3+^-ions are embedded into the host matrices with activators (viz., emitters Er^3+^, Ho^3+^, and Tm^3+^) and sensitizers (i.e., absorbers Yb^3+^). The majority of the host matrices are fluoride and oxide-based, viz., NaYF_4_, NaGdF_4_, LiYF_4_, BaYF_5_, Gd_2_O_3_, Y_2_O_3_, etc. [1]. Lanthanide (Ln)-doped UCNPs are a class of fluorescent imaging probes that have an edge over other luminous particles such as semiconductors, plasmonic nanoparticles, quantum dots, etc., due to their long and tunable emissions, minimal toxicity, and relatively high chemical and optical stability [2]. However, an array of constraints like hydrophobicity, toxicity, marginalized photochemical activity, brittleness under heat, potent photo-blinking, and impaired biological adaptability hinder their use in biomedical fields [3,4,5]. One significant challenge is their relatively low luminescence efficiency, which can limit their brightness and sensitivity in imaging applications. Additionally, the synthesis of UCNPs often requires high temperatures and toxic reagents, raising concerns about their biocompatibility and environmental impact. Furthermore, surface modification of UCNPs to enhance their stability and functionality in biological environments can be complex and may affect their optical properties. These limitations emphasize the need for continued research and development to optimize their performance for biomedical applications, especially in imaging [6,7,8,9,10]. Although NaYF_4_ is widely acknowledged as the optimal host matrix for upconversion processes, it does have certain drawbacks. The complex experimental setup or poor stability associated with organic components imposes new constraints in practical applications. NaYF_4_ contains two types of low-symmetry lanthanide sites, where the distorted electron cloud forms a strong coupling with the lattice, leading to the formation of numerous non-harmonic phonons, which hinders the energy transfer between the absorption and emission centers. On the other hand, in the cubic phase, Na^+^ and Ln^3+^ ions randomly compete with each other within the lattice, making the bonding more complex and resulting in even more non-harmonic phonon modes that cause detrimental energy loss channels, which are more pronounced than in the highly ordered cation distribution of the hexagonal lattice. The low quantum efficiency and the Laporte forbidden transition lead to significantly lower upconversion efficiency of NaYF_4_ or NaGdF_4_ [11,12,13]. In order to address the concerns, we considered CaF_2_ as a host matrix for embedding the lanthanides because of its high thermal and chemical durability, non-hygroscopic nature, and relative ease of synthesis [14,15,16]. Further, CaF_2_ has low vibrational energy, remarkable transparency across a broad wavelength range, and exhibits reduced potential for multi-phonon de-excitation [17,18,19,20,21,22]. 

Various chemical approaches have been established to fabricate the controlled morphology of UCNPs having desirable upconversion luminescent (UCL) properties. CaF_2_:Yb Er-based nanomaterials have been synthesized earlier; however, most of them involved high-temperature synthesis and large amounts of surfactant, due to which the UCNPs are hydrophobic and, hence, less suitable for bioimaging. For instance, Zhao et al. reported microwave-assisted synthesis of ATP-coated CaF_2_:Yb Er upconversion nanomaterials in an acidic medium and studied the effect of doping concentration of Yb/Er on the morphology, size, and crystallinity of the nanomaterials. However, the luminescence intensity was quite low even at a laser power of 0.5 W/cm^2,^ and therefore, the signal intensity of in vitro imaging of gastric carcinoma cells was low [23]. Yin et al. reported doping of CaF_2_:Yb, Er upconversion nanomaterials with alkali metals for enhancement of luminescence intensity [24]. Hence, it is desirable to find a method to synthesize chemically stable, water-dispersible, and highly luminescent UCNPs. The modified miniemulsion–coprecipitation method adopted in this work required a smaller amount of surfactant, and the reaction was carried out in aqueous media using a high-energy input method like high-pressure homogenization, which creates small droplets [25]. This method also enabled easy purification of excess surfactants and reactants. Sodium fluoride was used as a fluorine source, which is less toxic than the conventionally used ammonium fluoride [25,26]. The reproducibility rate of the miniemulsion-based coprecipitation method for preparing UCNPs can be higher due to the controlled microenvironment provided by the surfactant and co-surfactant systems. The homogenization step ensures uniform droplet formation, leading to consistent nucleation and growth of the nanoparticles across different batches. Additionally, the overnight precipitation step allows for complete and uniform particle formation, contributing to the reproducibility of the size, shape, and optical properties [27,28]. Further, the room temperature miniemulsion method has not yet been explored for synthesizing UCNPs that can be excited at 980 nm, as mentioned in Table 1. In this study, we aimed to enhance the luminescence of UCNPs for cellular imaging by systematically tuning both the activator concentration and the laser power. Specifically, we have varied the concentration of the activator (Er^3+^—2%, 2.5%, 3%, and 5%) and kept the sensitizer (Yb^3+^—20%) concentration constant to investigate its effect on luminescence intensity at low laser power. Also, the UCL (both red and green emission intensity) of the UCNPs can be tuned by varying the laser power (0.28 W–0.7 W) to see the effect of varying the laser power on the intensity of the luminescence. The UCNPs exhibited strong red emission under NIR light (980 nm), which is advantageous for cellular imaging due to the greater penetration depth of red light. According to the literature, this material typically displays enhanced green emission, with significant red emission observed only when doped with alkali metals [16,29,30]. The UCNPs also retained their red emission upon internalization into A549 and MCF-7 cancer cells, suggesting their potential for cellular imaging. The overarching goal of this project is to synthesize the UCNPs through the miniemulsion method so as to enhance the luminescence, which is crucial for imaging purposes. Additionally, we aimed to select a host material with low toxicity toward cancer cells, ensuring biocompatibility among available UCNPs. We also sought to utilize a synthesis method that allows for the production of UCNPs at room temperature in an aqueous medium, facilitating easier purification and broad applicability.

## 2. Results and Discussion

### 2.1. Structural and Vibrational Properties 

**Figure 1:**
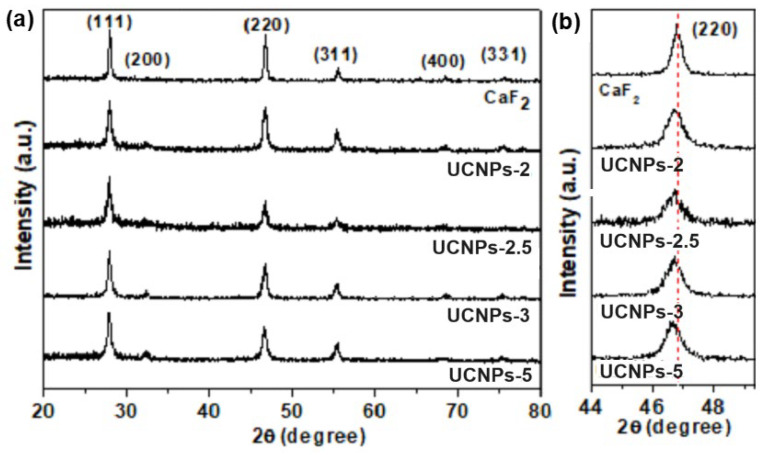
(**a**) XRD patterns of pure CaF_2_ and different UCNPs, and (**b**) shifting of the (220) diffraction peak towards the lower angle.

Figure 1 shows the (a) XRD patterns of pure CaF_2_ and different UCNPs and (b) shifting of the (220) diffraction peak towards a lower angle. The formation of highly crystalline cubic phase fluoride-type structure was confirmed by the presence of (111), (220), (311), (400), and (331) lattice planes in XRD patterns (Figure 1a) of both pure CaF_2_ and co-doped UCNPs (JCPDS Card no. 01-87-0976). The new diffraction peak appeared at a 2θ value of 32.84° in the XRD pattern of co-doped UCNPs, which can be attributed to the (200) lattice plane of the fluorite-type structure of CaF_2_. The appearance of this additional diffraction peak is mainly due to the successful incorporation of Yb^3+^ and Er^3+^ ions at the lattice site of Ca^2+^ [26]. Further, it has been observed that the dopant incorporation into the CaF_2_ host strongly affects the angular position and width of the diffraction peaks. The positions of the diffraction peaks shifted towards the lower angle gradually with the increased concentration of Er^3+^ dopant and simultaneously increased the peak broadening, which is clearly visible in the (220) diffraction peak shown in Figure 1b. This is mainly due to the gradual incorporation of F^-^ ions into the interstitial positions to compensate for the extra positive charge that is produced by the partial substitution of Ca^2+^ ions by Er^3+^ ions in the lattice [39]. 

The lattice constant (a), crystallite size, and lattice strain (**ε**) of CaF_2_ and co-doped UCNPs are given in Table S1. It has been found that the lattice constant of co-doped UCNPs increases with increasing Er^3+^ concentration due to the strong charge repulsion among the F^-^ ions. The decrease in crystallite size observed in co-doped systems is probably due to the development of lattice strain as a result of the occupation of dopant ions in the host lattice/alternation of host lattice packing [26]. Moreover, the absence of any impurity peaks associated with co-doping in XRD patterns of co-doped UCNPs indicates that the cubic phase fluorite-type structure of CaF_2_ was not affected upon the introduction of Yb^3+^ and Er^3+^ into the host lattice. 

**Figure 2:**
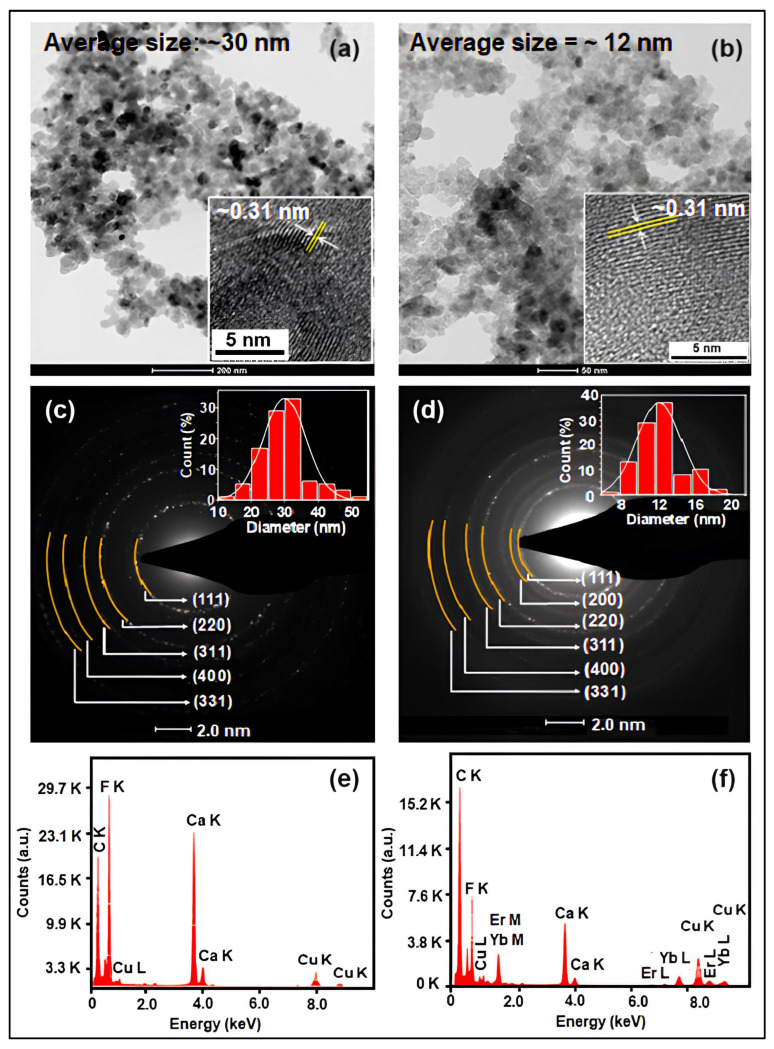
TEM micrographs of (**a**) pure CaF_2_ and (**b**) UCNPs-2 (inset: their HRTEM micrographs showing lattice spacing), SAED patterns of (**c**) pure CaF_2_ and (**d**) UCNPs-2 (inset: particle size distribution plots obtained from respective TEM micrographs of the figure), and TEM-EDS analysis of (**e**) pure CaF_2_ and (**f**) UCNPs-2.

Figure 2 shows the TEM micrographs of (a) pure CaF_2_ and (b) UCNPs-2 (inset: their HRTEM micrographs showing lattice spacing), SAED patterns of (c) pure CaF_2_ and (d) UCNPs-2 (inset: particle size distribution plots obtained from respective TEM micrographs), and TEM-EDAX analysis of (e) pure CaF_2_ and (f) UCNPs-2. TEM micrographs (Figure 2a,b) clearly showed the formation of well-spherical nanoparticles of average size of about 30 nm and 12 nm for CaF_2_ and co-doped UCNPs, respectively. The interplanar spacing of ~0.31 nm is observed for both pure CaF_2_ and UCNPs-2, which corresponds to the (111) plane of fluorite-type CaF_2_ [40]. The formation of highly crystalline single-phased fluorite-type structures is further supported by the presence of intense reflections from (111), (220), (311), (400), and (331) planes of CaF_2_ and (111), (200), (220), (311), (400), and (331) planes of co-doped UCNPs in their respective SAED patterns (Figure 2c,d). The appearance of an intense EDAX peak for Ca^2+^ and F^-^ in TEM-EDAX results of both pure CaF_2_ and UCNPs-2 clearly suggests the formation of the CaF_2_ host lattice (Figure 2e,f). Further, the successful doping of Yb^3+^ and Er^3+^ ions into UCNPs was confirmed by their presence in TEM-EDAX analysis. The weight % of Yb^3+^ and Er^3+^ was found to be 13.6 and 1.61 in UCNPs-2, which indicates that the amount of lanthanide ions incorporated into the CaF_2_ host is less than the actual amount of dopants added during the synthesis. Moreover, the absence of any additional peaks suggested the formation of contaminant-free CaF_2_ and co-doped UCNPs. The vibrational phonon modes of pure CaF_2_ and co-doped UCNPs were investigated by Raman spectroscopy to identify the distortions induced in the crystal lattices upon doping (Figure S1, Supplementary data). The distinctive phonon vibrational band observed at 321 cm^−1^ in the Raman spectrum of pure CaF_2_ can be ascribed to the T_2g_ mode arising due to the oscillation of fluorine ions towards each other [41]. However, the most intense phonon mode of UCNPs-2 appeared at 255 cm^−1,^ along with other modes at 405, 585, and 670 cm^−1^. These phonon modes may be attributed to the perturbations that arise due to doping of the CaF_2_ host matrix with Yb/Er [42,43].

**Figure 3:**
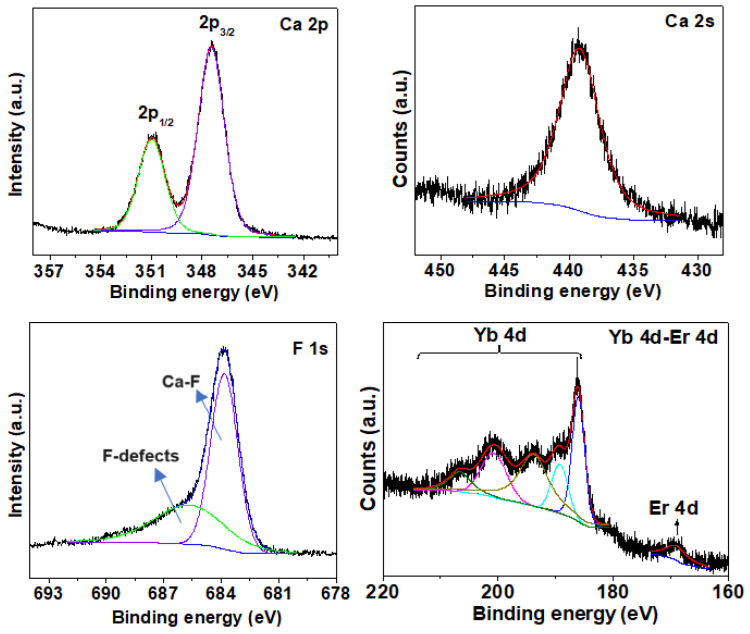
High-resolution XPS spectra of Ca 2p, Ca 2s, F 1s, and Yb 4d-Er 4d of UCNPs-2.

XPS analysis was employed to determine the oxidation state of the elements and to identify their chemical environment. An XPS survey scan performed on UCNPs showed the presence of Ca, F, Yb, and Er other than adventitious C. Figure 3 shows the high-resolution XPS spectra of (a) Ca 2p, (b) Ca 2s, (c) F 1s, and (d) Yb 4d–Er 4d of UCNPs-2. The Ca 2p peak can be resolved into two peaks centered at 347.4 and 350.96 eV, corresponding to Ca 2p_3/2_ and Ca 2p_1/2_ with spin–orbit splitting of 3.5 eV and area ratio of 1:2. This suggests the presence of Ca ions in the +2 oxidation state, which was further supported by the appearance of a highly symmetrical Ca 2s spectrum centered at 439.2 eV [44,45]. The F 1s spectrum can also be deconvoluted into two components centered at 683.83 and 685.67 eV, indicating the presence of F ions in two different chemical environments. The peak-centered lower binding energy can be ascribed to Ca-F bonding (lattice F^−^ ions), whereas the higher binding energy peak can be associated with F-defects in CaF_2_ (F^−^ ions in interstitial positions) [46]. The presence of two different types of F^-^ ions further supports the findings of XRD analysis (i.e., shifting of diffraction peaks towards a lower angle with the incorporation of F^-^ ions into the interstitial positions). The Yb 4d spectrum is found to be quite complicated as the Yb^3+^ oxidation level is highly affected by strong Coulomb interaction. As a result of this interaction, the Yb 4d peak split into five peaks centered at around 184.7, 186.0, 186.2, 193.8, 200.7, and 206.6 eV, corresponding to Yb^3+^ oxidation [47]. Further, the appearance of an Er 4d peak centered at 168.9 eV confirmed the presence of Er in +3 oxidation states in UCNPs [48]. From XPS studies, it has been observed that Yb and Er exist in the form of Yb^3+^ and Er^3+^ in doped UCNPs.

### 2.2. NIR Absorbance and Photoluminescence (PL) Characteristics

The absorbance study was performed to confirm that the absorption band of developed UCNPs lies around 980 nm. The resulting spectrum of UCNPs-2 has shown a strong absorption band with maxima at around 975 nm, which mainly arises due to the spin-allowed transition of ^2^F_7/2_ to ^2^F_5/2_ of Yb^3+^ ions (Figure S2, Supplementary data). Thus, the developed UCNPs can be excited efficiently by a 980 nm laser for upconversion applications. The photoluminescence study was also performed to explore the upconversion behavior of the synthesized materials when excited with NIR light at 980 nm using a fiber-optic coupled diode laser. It is reported that the large overlap of the ^2^F_5/2_ →  ^2^F_7/2_ emission line of sensitizer (Yb^3+^ ion) and ^4^I_15/2_ →  ^4^I_11/2_ emission transitions of activator (Er^3+^ ions) leads to efficient energy transfer between sensitizer and activator ion in a single crystal that can effectively enhance the luminescent performance of a material [49]. Er^3+^ ions in the ^4^I_11/2_ state relax to the ^4^I_13/2_ state and are then stimulated to the ^4^F_9/2_ state by phonon-assisted energy transfer, as opposed to being directly excited to the ^4^F_7/2_ state. To capitalize on an effective energy transfer upconversion (ETU), a sensitizer with a large absorption cross-section in the NIR spectrum is typically co-doped with the activator to increase UCL efficiency [42]. The Yb^3+^ transition between ^2^F_7/2_ and ^2^F_5/2_ is highly resonant with f → f transitions of common upconverting Ln^3+^ ions, such as Er^3+^, facilitating effective energy transfer from Yb^3+^ (sensitizer) to Er^3+^ (activator). 

**Figure 4:**
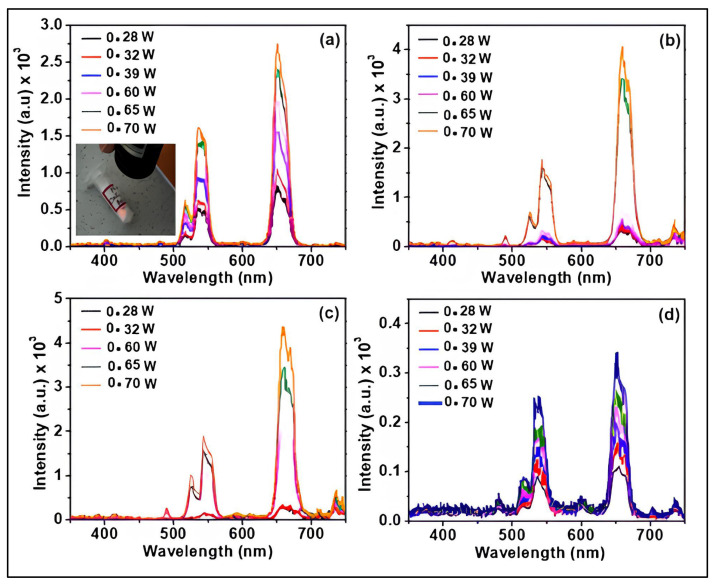
PL spectra of (**a**) UCNPs-2, (**b**) UCNPs-2.5, (**c**) UCNPs-3, and (**d**) UCNPs-5 under irradiation of 980 nm laser light. Inset of Figure 4a shows the photograph of revealing red emission arising from UCNPs-2 under irradiation of 980 nm laser light.

Figure 4 shows the PL spectra of (a) UCNPs-2, (b) UCNPs-2.5, (c) UCNPs-3, and (d) UCNPs-5. As mentioned before, lanthanides are associated with the filling of the 4f shell, so they are well-suited for upconversion applications due to the existence of multiple metastable levels. Er^3+^ is frequently used as an activator because of its typical feature of ladder-like arranged energy levels. Among the Ln^3+^ ions, Yb^3+^ has been considered an efficient absorber/sensitizer ion because of its higher absorption coefficient (1.16  ×  10^–20^ cm^2^) and simple electronic configuration. It is well-reported that the co-doped CaF_2_:Yb^3+^, Er^3^ UCNPs exhibited the most intense emission bands in the green (centered at 520 nm due to ^2^H_11/2_ →  ^4^I_15/2_ and ^4^S_3/2_ →  ^4^ I_15/2_ transitions) and red spectral region (centered at 660 nm due to ^4^F_9/2_ →  ^4^I_15/2_ transition), accompanied by the albeit very weak blue band (centered at 480 nm due to ^4^F_7/2_ →  ^4^ I_15/2_ transition) and a weak red band at 700 nm (due to ^4^F_7/2_ →  ^4^ I_13/2_ transition). The earlier findings also showed that while Ln^3+^ cations occupy crystal lattice positions with lower point symmetry, the as-synthesized CaF_2_:Er^3+^, Yb^3+^ nanomaterials have the cubic structure with a space group of Fm3m [30]. This could also result in high upconversion efficiency when exposed to a 980 nm diode laser. Furthermore, the CaF_2_ host material, with its cubic crystal structure and high symmetry, provides a stable and uniform environment for dopant ions. This high symmetry typically results in minimal splitting of energy levels, which can lead to sharp and well-defined emission lines. The relatively low phonon energy of CaF_2_ also helps reduce non-radiative losses, enhancing the overall luminescence efficiency. In the present study (from Figure 4), it has been observed that the emission spectrum of the developed UCNPs mainly exhibited two well-predominant emissions: ^4^F_9/2_ transition in the red region and ^4^S_3/2_ transition in the green region. As suggested in the literature, these two dominant emission transitions occur by a two-photon process in accordance with the power-dependent study [50]. The UCL intensity of these two bands was found to be strongly dependent on the concentration of Er^3+^ ions and applied laser power. The UCL intensity increases continuously with increasing the Er^3+^ ion concentration from 1 to 3 mol% upon irradiation of 980 nm laser light of different laser power. However, it decreases as the doping concentration exceeds 3 mol% gently because the cross-relaxation energy transfer enhances remarkably, resulting in a non-radiative depopulation of the excitation energy. The strong upconversion emission in the co-doped UCNPs can also be attributed to the clustering of lanthanide ions within the lattice, which enhances energy transfer between Yb^3+^ and Er^3+^. Boulon et al. observed this clustering in Yb^3+^-doped CaF_2_ single crystals. However, when the dopant concentration exceeds 3%, clustering can also lead to concentration quenching, reducing the emission efficiency [32,34]. Energy migration between dopant ions becomes increasingly significant as the concentration of Er^3+^ ions rises. At higher concentrations, the close proximity of these ions allows excitation energy to transfer from one ion to another, which can lead to quenching if the energy is transferred to an ion in a non-radiative state or if cross relaxation occurs, where two nearby ions exchange energy in a way that leads to a loss of excitation. This is crucial in determining the overall luminescence efficiency of UCNPs, especially at higher concentrations. It is noteworthy that even at a relatively low laser power of 0.28 W, the developed UCNPs exhibit significant UCL characteristics, demonstrating the high efficiency of the energy transfer process within the material. As the laser power is gradually increased from 0.28 W to 0.7 W, there is a simultaneous enhancement in the intensities of both green and red emissions. This indicates that the luminescent output can be finely tuned by adjusting the laser power, providing flexible control over the emission profile. Additionally, by varying the concentration of Er ions in the UCNPs, the ratio of green to red emissions can be further optimized, allowing for precise modulation of the luminescence properties to meet specific application needs. This dual approach that manipulates both dopant concentration and laser power density enables the development of UCNPs with customizable optical properties, making them highly versatile for various biomedical applications.

From the log–log plots (Figure S3, Supplementary Data), it has been found that the relation between intensity and laser power follows a linear relationship within the studied excitation power density range. Thus, it can be concluded that both green and red emissions follow a two-photon excitation path as the n value lies between 1.5 and 2.5. Further, the dominance of red emission arising from UCNPs under irradiation of 980 nm laser light over green is evident from the photograph provided in the inset of Figure 4a.

**Figure 5:**
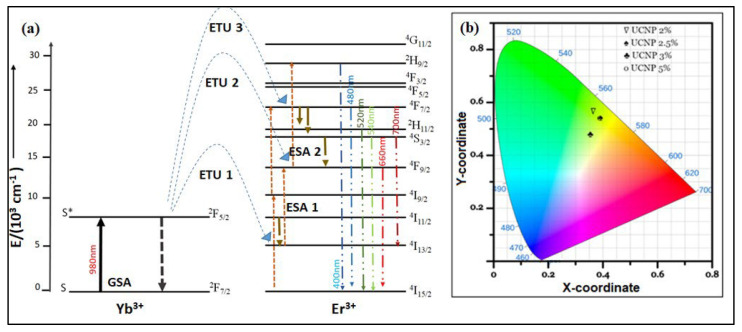
(**a**) Energy level diagram of co-doped CaF_2_:Yb^3+^, Er^3+^ nanoparticles solid; dotted and dashed-doted arrows represent photon absorption or emission, energy transfer, and relaxation processes, respectively; and (**b**) CIE chromaticity diagram showing color coordinates.

Figure 5a shows the energy level diagram of co-doped CaF_2_:Yb^3+^, Er^3+^ nanoparticles (solid, dotted, and dashed-doted arrows represent photon absorption or emission, energy transfer, and relaxation processes, respectively). Additionally, Figure 5b Commission Internationale de l’Eclairage (CIE) chromaticity diagram shows color coordinates where the X-coordinate represents the proportion of red color and the Y-coordinate represents the proportion of green color. All the transitions, viz., ^4^S_3/2_, ^2^H_11/2_, ^4^F_9/2_ →  ^4^I_15/2_ (red and green emission), result from the initial population of ^4^I_15/2_ →  ^4^I_9/2_ and ^4^I_9/2_ →  ^4^F_7/2_ for red emission, and non-radiative decay from ^4^F_7/2_ to ^4^S_3/2_ for green emission (Figure 5a). Non-radiative decay from ^4^S_3/2_ or ^2^H_11/2_ may directly populate ^4^F_9/2,_ resulting in red emission [42,51]. Non-radiative decay from ^4^I_11/2_ or radiative decay from ^4^S_3/2_ both populate ^4^I_13/2_, which can then be excited to ^4^F_9/2_ by energy transfer from a nearby Yb ion. The population of energy increases at ^4^F_9/2_, which depends on the population of ^4^I_13/2,_ and hence, the intensity of red emission is brighter at the time of energy transfer from Yb^3+^ to Er^3+^. Due to the maximum population at ^4^F_9/2_, a bright red color luminescence in the particle and a weak green emission can be seen due to the comparatively lesser population at ^4^S_3/2_ [52]. The x, y color coordinates were determined to validate the visible green and red emissions of different concentrations of Er^3+^ in CaF_2_:Yb, Er UCNPs using the CIE chromaticity diagram (CIE = Commission Internationale de l’Eclairage) (Figure 5b). The upconversion nanocrystals of different concentration coordinates in the green region of CIE chromaticity diagram ^2^H_11/2_ →  ^4^I_15/2_ and ^4^S_3/2_ →  ^4^I_15/2_ transitions of the Er^3+^. Here, we could observe that as the intensity of red is increasing with the increase in the power density, there is a simultaneous increase in the green emission as the calculated green-to-red ratio is constant. Hence, the CIE chromaticity falls towards the light yellow region, possibly due to the combination of both green and red-colored luminescence.

**Figure 6:**
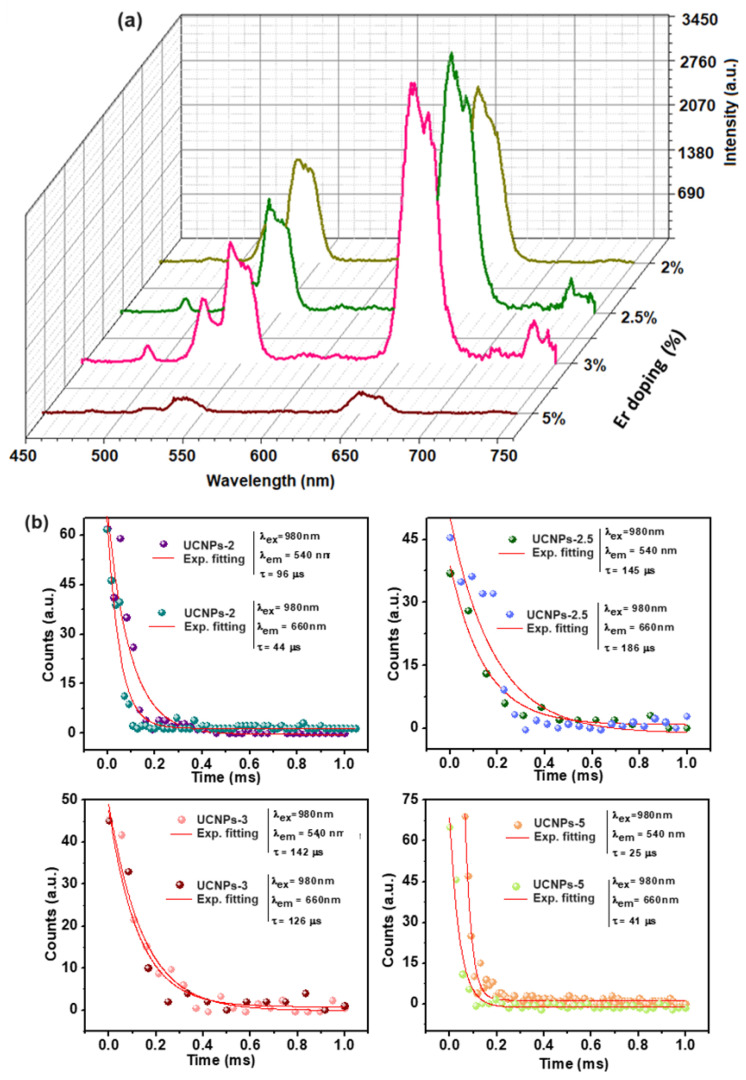
(**a**) Influence of varying amounts of Er^3+^ on UCL intensity of UCNPs and (**b**) decay kinetics for green (λ_em_ = 50 nm) and red (λ_em_ = 660 nm) emission.

Decay studies were performed to investigate the radiative lifetime of the photons at their energy level. Figure 6 shows the (a) influence of varying amounts of Er^3+^ on UCL intensity and (b) decay kinetics for green (λ_em_ = 540 nm) and red (λ_em_ = 660 nm) emissions of UCNPs. Since the Er^3+^ ions on the surface and in the bulk phase of the particles are affected by their surroundings differently, the underlying decay kinetics are complicated. Er^3+^ ions close to the nanoparticle surface may be responsible for the observed short luminescence decay time, but Er^3+^ ions in the bulk phase of the nanoparticles may be the cause of the lengthy luminescence decay time [42]. There is an increase in UCL intensity with an increase in Er^3+^ up to 3 mol%. However, it decreases with a further increase in Er^3+^ concentration (beyond 3 mol%) (Figure 6a). This is possibly due to concentration quenching caused by non-radiative energy transfer and cross-relaxation between Er^3+^ ions. The decay curves (Figure 6b) also align with the above observation, showing the dependence of UCL on the Er^3+^ concentration. Even though the decay data are weak and the number of data points is less, they can be fitted with an exponential factor, and a lifetime can be evaluated from it. The τ value at 660 nm (red emission) was more than that of the τ value at 540 nm (green emission) due to the maximum population of photons at ^4^F_9/2_. A slight decrease in the τ value at a doping concentration of 3 mol% could possibly be due to the initiation of the quenching process [10]. However, even at a low τ value, the UCL intensity was sufficient to image the cancer cells (discussed in Section 3.3). Further, the decay studies give an overview of the temporal response of the upconversion process with respect to the excitation rather than the intrinsic lifetime of the luminescence-emitting state [53].

### 2.3. Light Scattering and Upconversion Luminescence Imaging Properties

The colloidal stability of UCNPs in an aqueous medium is an important parameter for biological applications of UCNPs. From DLS measurements, the average intensity-weighted hydrodynamic diameter of UCNPs-2 was found to be ~160 nm (Figure S4, Supplementary data). The observed higher average hydrodynamic diameter obtained by DLS than that by TEM can be attributed to the polydispersity in size (d) of the particles and the d^6^ dependence of the scattering intensity on particle size [54]. In addition, self-aggregation of UCNPs may also contribute to the observed higher diameter by DLS. It has been observed that the UCNPs are colloidally stable in aqueous medium for about 24 h. Zeta-potential measurements showed the pH-dependent surface charge of UCNPs features (Figure S5, Supplementary Data). The UCNPs-2 possesses a positive charge at low pH and a negative change in the basic medium with an isoelectric point at 6.7. The pH-dependent surface properties of UCNPs can be explained by the adoption characteristics of H^+^ and OH^−^ ions on their surface. However, the absolute zeta potential of UCNPs is very low to provide long-term colloidal stability to them. 

**Figure 7:**
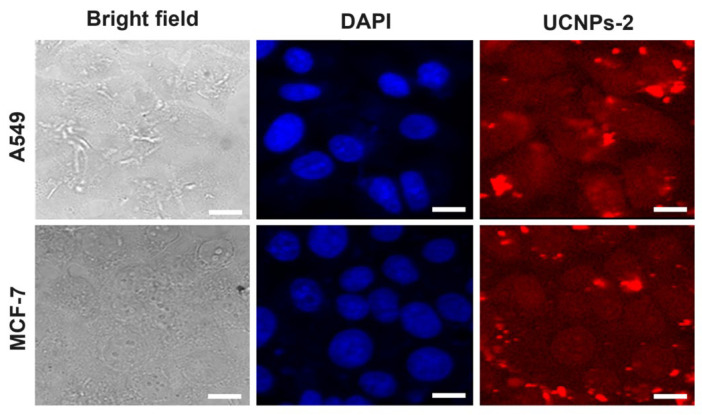
Confocal microscopy images of A549 and MCF-7 cells after overnight incubation with UCNPs-2 under 980 nm laser light irradiation (Scale bar: 10 µm).

Further, the developed UCNP powder exhibited strong red emission under irradiation of 980 nm light (Figure S6, Supplementary Materials). This notable red emission prompted us to explore the cellular imaging capability of these UCNPs under NIR irradiation, leveraging the deep tissue penetration and reduced photodamage associated with NIR light. Figure 7 shows the confocal microscopy images of A549 and MCF-7 cells after overnight incubation with UCNPs-2 under 980 nm laser light irradiation. The blue-color fluorescence emission arises from the DAPI-stained nuclei. A substantial uptake of UCNPs-2 by both A549 and MCF-7 cells was evident from the strong red color fluorescence emission, which indicates their successful internalization. The successful internalization of UCNPs into cells is crucial for effective cellular imaging, as it ensures that the UCNPs are sufficiently distributed within the cells to provide clear and detailed images. However, a few chunks of UCNP-2 were also observed adhering to the cell surface (bright clustered fluorescent signal) in the present study, which can be overcome by suitable surface modification. The red emission of UCNPs-2 under NIR light is particularly advantageous compared to the commonly observed green emission in other studies, as red light offers deeper tissue penetration and lower background interference, making it more suitable for in vivo imaging applications. Though these UCNP-2 exhibited dose-dependent toxicity towards cancer cells after their 24 h incubation under culture conditions (Figure S7, Supplementary Data), they would not cause significant toxicity within the treatment time (18 h) of cellular imaging. Moreover, the surface functionalization of these developed UCNPs with suitable ligands/materials will be performed in the future to further improve their long-term colloidal stability and biocompatibility. In comparison to previous studies, our research highlights the unique advantage of strong red emission of CaF_2_-based UCNPs under NIR light and their potential use in cancer cell imaging with the added benefit of lower toxicity for short-term applications. Specifically, these findings underscore the significance of our research in advancing the field of luminescent bioimaging and offer valuable insights for future developments in nanoparticle-based imaging technologies. 

## 3. Experimental Section

### 3.1. Materials and Methods

Calcium nitrate tetrahydrate (Ca(NO_3_)_2_·4H_2_O), ytterbium nitrate (Yb(NO_3_)_3_·5H_2_O), erbium nitrate (Er(NO_3_)_3_·5H_2_O), sodium fluoride (NaF), Igepal^®^ CO-630, Brij^®^ 52, and cyclohexane were procured from Sigma Aldrich, St. Louis, MO, USA. Deionized water was obtained from a water purification system (Smart-S30, Hitech, Shanghai, China). All the chemicals were of analytical grade and were used without any additional purification. 

### 3.2. Synthesis of Co-Doped CaF_2_:Yb^3+^, Er^3+^ UCNPs

**Scheme 1:**
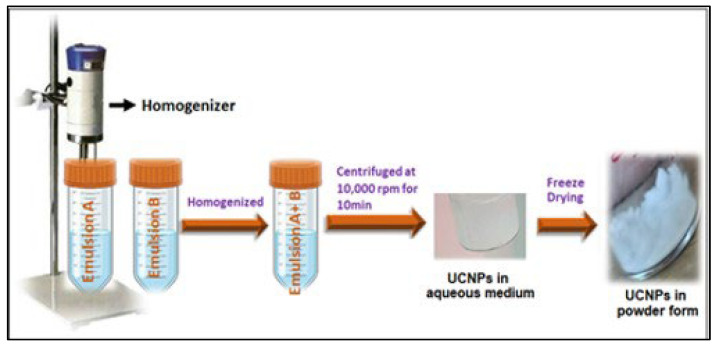
Schematic representation of synthesis of CaF_2_ and CaF_2_:Yb^3+^, Er^3+^ UCNPs.

The co-doped CaF_2_:Yb^3+^ (20%), Er^3+^ (2%, 2.5%, 3%, and 5%) UCNPs were synthesized using a modified miniemulsion-mediated coprecipitation method [25]. In a typical synthesis (Scheme 1), two separate dispersions, A and B, were prepared by dispersing 200 mg of Igepal^®^ CO-630 and 600 mg of Brij^®^ 52 in 8 mL of cyclohexane. Subsequently, 5 mL of 0.25 M Ca(NO_3_)_2_.4H_2_O were added to dispersion A and homogenized for 5–7 min at 10,000 rpm, yielding emulsion A, while 5 mL of aqueous solution of 1 M NaF was added to dispersion B and homogenized to form emulsion B. Both the emulsions were mixed and homogenized for 5–7 min at 10,000 rpm, forming pure CaF_2_ nanoparticles. The suspension was allowed to stand overnight and was separated by centrifugation (10,000 rpm for 10 min) and washed with deionized water to remove the surfactants. The procedure of centrifugation and washing was repeated till the surfactants were removed. The co-doped systems were prepared using a similar protocol with the addition of 20% (0.25 mmol) of Yb(NO_3_)_3_·5H_2_O and different concentrations of Er(NO_3_).6H_2_O (2%, 2.5%, 3%, 5%, i.e., 0.025 mmol, 0.03 mmol, 0.035 mmol, and 0.0625 mmol, respectively) to the Ca-containing solution. For brevity, the co-doped samples CaF_2_: Yb^3+^ (20%) with 2%, 2.5%, 3%, and 5% Er^3+^ are named as UCNPs-2, UCNPs-2.5, UCNPs-3, and UCNPs-5, respectively, based on the concentration of Er used in their preparation. 

### 3.3. Characterization

Powder X-ray diffraction (XRD) of the UCNPs was recorded on a Rigaku diffractometer using copper Kα at an operating voltage of 40 kV and 30 mA. The crystallite size and lattice strain were determined on the X-ray line broadening of the peaks at the (111), (220), and (311) planes using the following equations:

$$Crystallite\;size\;(D)=\frac{0.89\;\lambda }{\beta \;cos\;\theta }$$

$$\;Lattice\;strain\;(\epsilon )=\frac{\beta }{4\;tan\;\theta }$$

where *λ* is the X-ray wavelength used, *β* is the angular line width at half maximum intensity, and *θ* is the Bragg’s angle. The lattice constant (a) was calculated using FullProf programming. TEM analysis was performed on a 300 kV field emission gun-transmission electron microscope (FEG-TEM 300 kV, FEI Tecnai G2, Hillsboro, OR, USA) equipped with EDAX (Octane ELITE T70. The photoluminescence (PL) spectra were taken at different laser powers (0.28 W to 0.70 W) using an Edinburgh Instrument FLS920 (Kirkton Campus, UK) attached to a 980 nm CW laser. For PL studies, 2–3 mg of samples were drop-cast on a glass slide by dispersing in ethanol medium and dried thoroughly for complete removal of solvent. The UV–visible spectra of UCNPs were recorded on a Shimadzu spectrophotometer. Raman spectra were recorded using a STR-300 micro-Raman spectrometer (SEKI Technotron, Tokyo, Japan) at an excitation wavelength of 532 nm. X-ray photon spectroscopy (XPS) studies were performed on UCNPs-2 using an ESCALAB 250Xi (Thermo Fisher Scientific, Waltham, MA, USA) equipped with a monochromatized X-ray source producing Al Kα radiation (hν = 1486.6 eV) from an Al anode. For obtaining a good resolution of each element, a pass energy of 10 eV was chosen. As an internal reference for the absolute binding energy, the C-1s peak (284.6 eV) was used. The deconvolution of the peaks was performed with Lorentzian–Gaussian line shapes using the XPSPEAK (Version 4.1) software after performing the Shirley background subtraction. The intensity-weighted hydrodynamic diameter was measured using a Malvern 4800 Autosizer, Zetasizer Nano series (Malvern Panalytical, Malvern, Worcestershire, UK)mploying a 7132-digital correlator with a He–Ne laser light source having a power output of 15 mW. The zeta potential of UCNPs was recorded on the Zetasizer nanoseries from Malvern Instruments (Malvern Panalytical, Malvern, Worcestershire, UK). Confocal microscopy imaging was performed on UCNP-treated A549 and MCF-7 cells with 980 nm excitation. For imaging, cells (1 × 10^6^) were seeded on glass coverslips and cultured overnight. The cells were then treated overnight with UCNPs-2 at a concentration of 250 µg/mL under culture conditions, followed by washing with PBS 3–4 times. After washing, cells were fixed with 4% paraformaldehyde and permeabilized using 0.1% triton X. The cells were then mounted on a glass slide in cell mounting medium (Invitrogen, Thermo Fisher Scientific, Waltham, MA, USA) containing DAPI for nuclear staining and then imaged by confocal microscopy (Zeiss LSM 780 confocal/multiphoton microscope, Oberkochen, Germany) using a red filter for UCNPs luminescence and a blue filter for DAPI. 

## 4. Conclusions

Highly luminescent CaF_2_:Yb^3+^/Er^3+^ UCNPs were prepared by the miniemulsion-mediated coprecipitation method at room temperature. This approach not only simplifies the synthesis process without complex surface engineering but also ensures easy purification of the nanomaterials, making it a cost-effective and scalable method for producing UCNPs for imaging applications. The XRD and TEM analyses revealed the formation of crystalline, single-phase cubic fluorite-type nanostructures. XPS and EDAX analyses confirmed the successful doping of Yb^3+^ and Er^3+^ into the CaF_2_ matrix, and both exist in the +3 oxidation state. Remarkably, the UCNPs exhibited strong upconversion luminescence with distinct emission peaks in the visible region (red and green), even at low laser power (0.28 W). It has been found that the luminescence characteristics increase with increasing the Er^3+^ concentration up to 3 mol%. Due to reduced quenching and weak non-radiative emission, CaF_2_:Yb^3+^/Er^3+^ nanocrystals boost the red color intensity appreciably by back-energy transfer to Er^3+^. It is noteworthy to mention that the UCNPs developed in the present study showed good luminescence behavior without the formation of core–shell structures or additional surface alternations, which are often required in other systems but can reduce luminescence due to surface quenching effects. Further, these nanoparticles retained their red luminescence characteristics upon internalization into the cancer cell lines, suggesting their potential capability for cellular imaging. Specifically, they will serve as excellent luminescent probes for cellular imaging under NIR irradiation upon enhancement of long-term colloidal stability and biocompatibility.

## Figures and Tables

**Table 1:** Overview of UCNPs: Materials, dopants, emissions, synthesis methods, and conditions.

Sr. No.	Host Material	Dopant	Ratio	Emission	Synthesis Method	Laser Power	Stabilizing/Capping Agent	Reference
1.	CaF_2_	Yb, Er, Mn	15%, 2%, (0, 0.7, 2.6, 4.7, 6 and 7)	Red and green varied upon Mn concentration	In situ hydrothermal condition	-	-	[31]
2	CaF_2_	Yb, Er	20%, 2%	Green and red	One pot hydrothermal	-	Oleate ions	[32]
3	CaF_2_	Er	0.05–1%	Red and green	Temperature gradient method	-	-	[33]
4	CaF_2_	Yb/Er	20%, 2%	Red and green	Hydrothermal, coprecipitation, and decomposition	10–80 W cm^−2^	Tri-sodium citrate	[34]
5	LiYF_4_	Yb, Er	3%, 0.025%	Green (temp tuned)	Thermal decomposition	65–250 W cm^−2^	Cysteine	[35]
6	NaYF_4_	Yb, Er	20%, 2%	Strong Blue and Red	PVP-assisted method	1300 W	PVP	[36]
7	NaYF_4_	Yb, Er	(20–100%), 2%	Green and red	Thermal decomposition	7 W cm^−2^ and 50 mW mm^−2^	Oleate	[37]
8	NaYF_4_	Yb, Er, and Nd	20, 2, 10	Green and red	Thermal decomposition	13 W cm^−2^	-	[38]
9	CaF_2_	Yb, Er	20%, (2%, 2.5%, 3% and 5%)	Red and green (red dominant)	Miniemulsion-mediated coprecipitation method	0.28 W–0.7 W	-	This work

## Data Availability

The original contributions presented in the study are included in the article/Supplementary Materials, further inquiries can be directed to the corresponding author/s.

## Supplementary Materials

The following supporting information can be downloaded at: https://www.mdpi.com/article/10.3390/molecules29174177/s1, Figure S1. Raman spectra of pure CaF2 and co-doped UCNPs-2. Figure S2. Absorption spectra of UCNPs-2 showing strong absorption in NIR region. Figure S3. ln(I) vs. ln (P) plots of (a) UCNPs-2, (b) UCNPs-2.5, (c) UCNPs-3 and (d) UCNPs-5 recorded at two different emission wavelengths (540 nm and 660 nm) showing power dependent PL characteristics. Figure S4. Intensity weighted hydrodynamic diameter of UCNPs-2 at 0 and 24 h. Figure S5. pH dependent zeta-potential of UCNPs-2. Figure S6. Photograph showing red emission from UCNPs-2 powder under 980 nm light irradiation (the image was taken by mounting UCNPs powder under Zeiss LSM 780 confocal/multiphoton microscope). Figure S7. Viability of A549 and MCF-7 cells upon incubated for 24 h with UCNPs-2. Table S1. The lattice constant (a), crystallite size and lattice strain (ε) of CaF2 and co-doped UCNPs.
