# Supplementary material for: Maximizing Upconversion Luminescence of Co-Doped CaF₂:Yb, Er Nanoparticles at Low Laser Power for Efficient Cellular Imaging
Source: Molecules. 2024 Sep 3;29(17):4177. doi: 10.3390/molecules29174177 (PMC11397371; doi:10.3390/molecules29174177)
Supplement: Supplementary file 1 [file molecules-29-04177-s001.zip › molecules-3177612-supplementary.pdf]

Maximizing Upconversion Luminescence of co-doped CaF_2:Yb, Er Nanoparticles at Low Laser Power for Efficient Cellular Imaging

Neha Dubey[1,2], Sonali Gupta[2,3], S. B. Shelar[2], K. C. Barick[2,3,*], Sudeshna Chandra[4,*]

[1]*Department of Chemistry, Sunandan Divatia School of Science,*
SVKM's NMIMS (deemed to-be) University, Mumbai-400056, India
[2]*Chemistry Division, Bhabha Atomic Research Centre, Trombay, Mumbai – 400 085, India*
[3]*Homi Bhabha National Institute, Anushaktinagar, Mumbai – 400 094, India*
[4]*Hanse-Wissenschaftskolleg – Institute for Advanced Study (HWK)*
Lehmkuhlenbusch 4, 27753 Delmenhorst, Germany

**Corresponding authors: kcbarick@barc.gov.in (K.C. Barick),*
sudeshna.chandra@uni-oldenburg.de (S. Chandra)

Supplementary data

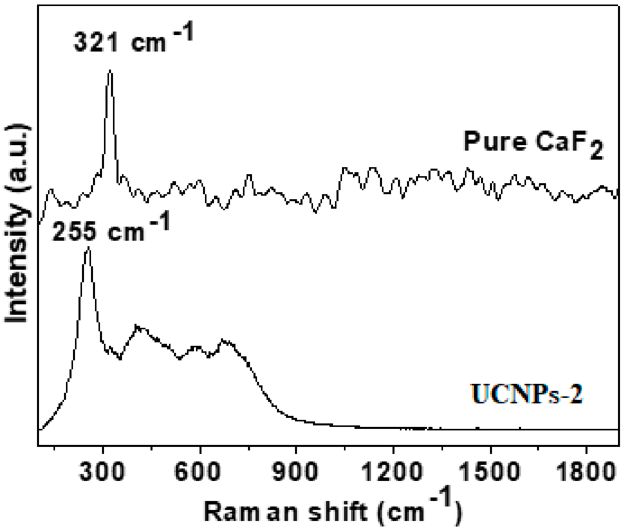

Figure S1. Raman spectra of pure CaF_2 and co-doped UCNPs-2.

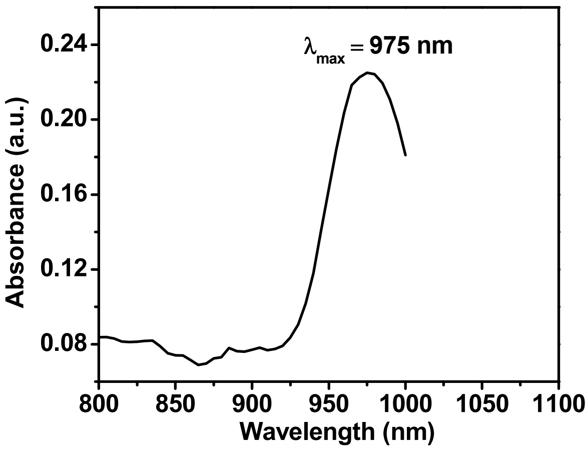

Figure S2. Absorption spectra of UCNPs-2 showing strong absorption in NIR region.

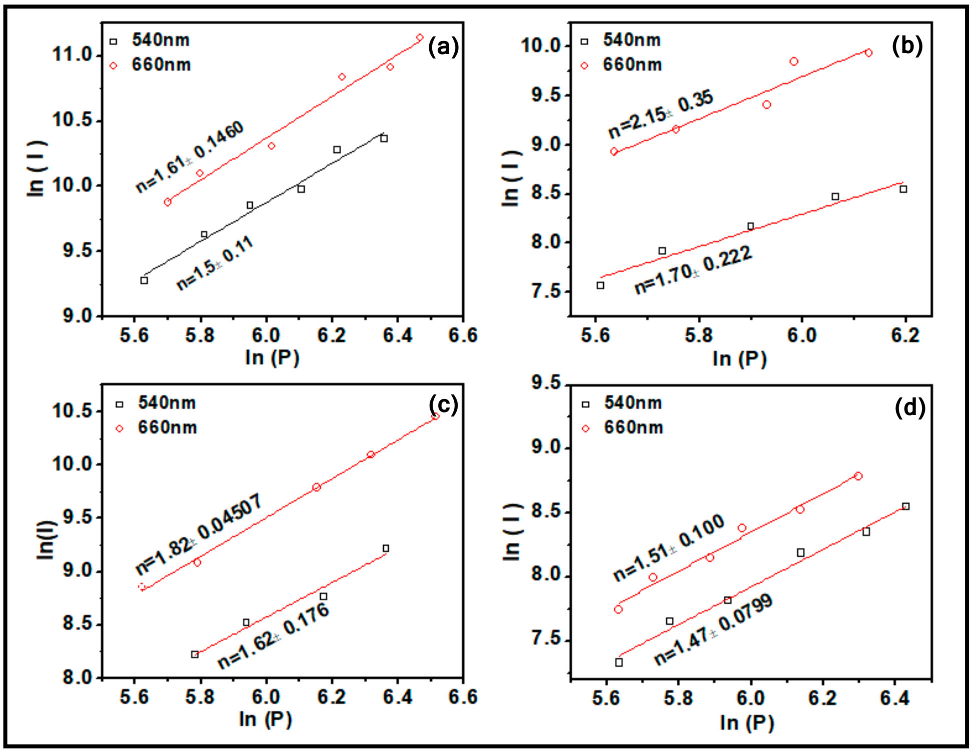

Figure S3. ln(I) vs. ln (P) plots of (a) UCNPs-2, (b) UCNPs-2.5, (c) UCNPs-3 and (d) UCNPs-5 recorded at two different emission wavelengths (540 nm and 660 nm) showing power dependent PL characteristics.

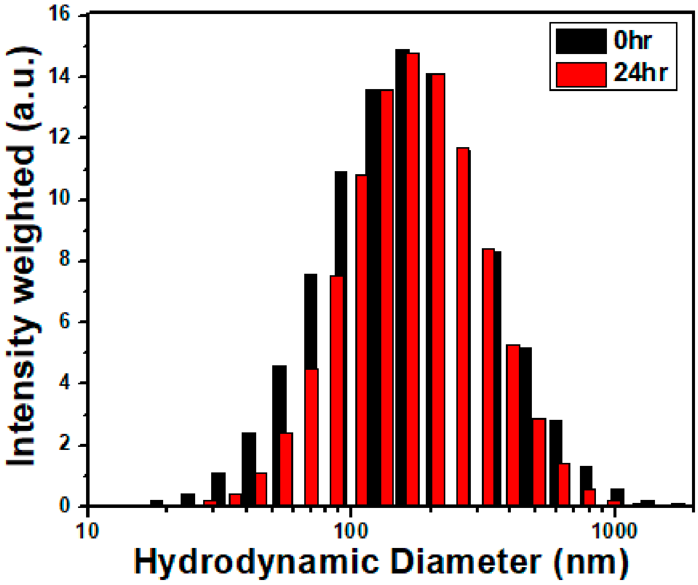

Figure S4. Intensity weighted hydrodynamic diameter of UCNPs-2 at 0 and 24 h.

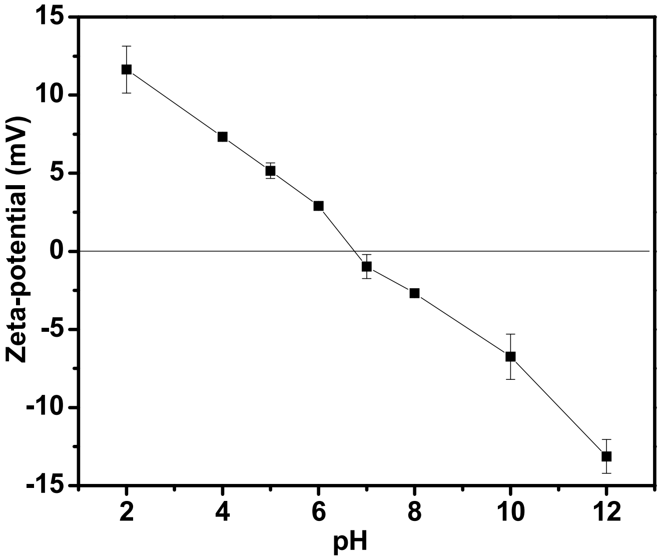

Figure S5. pH dependent zeta-potential of UCNPs-2.

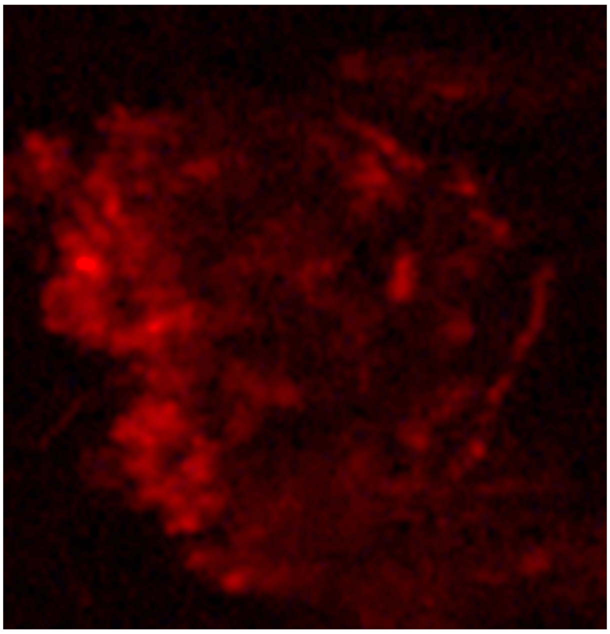

Figure S6. Photograph showing red emission from UCNPs-2 powder under 980 nm light irradiation (the image was taken by mounting UCNPs powder under Zeiss LSM 780 confocal/multiphoton microscope).

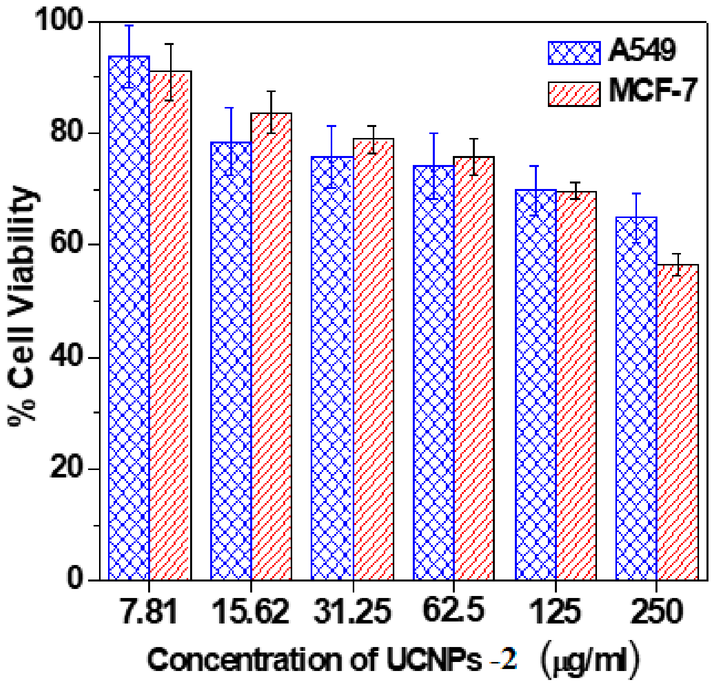

Figure S7. Viability of A549 and MCF-7 cells upon incubated for 24 h with UCNPs-2.

Table S1. The lattice constant (a), crystallite size and lattice strain (ε) of CaF_2 and co-doped UCNPs.

**Samples**	**CaF_2**	**UCNPs-2**	**UCNPs-2.5**	**UCNPs-3**	**UCNPs-5**
**Crystallite size (nm)**	21.7	14.4	13.6	13.3	12.9
**Lattice parameter (Å)**	5.4850	5.4924	5.4949	5.4971	5.5037
**Lattice strain**	0.0047	0.0073	0.0080	0.0087	0.0099
